# Effect of the Cytokinin Type in the Culture Medium on the Ultrastructure of Leaf Chloroplasts and Photosynthetic Pigment Content of In Vitro Apple (*Malus x domestica* Borkh.) Shoots

**DOI:** 10.3390/plants15020223

**Published:** 2026-01-11

**Authors:** Zsuzsa Máthéné Szigeti, Katalin Solymosi, Richárd Kovásznai-Oláh, Judit Dobránszki

**Affiliations:** 1 Center of Agricultural Genomics and Biotechnology, Faculty of Agriculture and Food Sciences and Environmental Management, University of Debrecen, 4400 Nyíregyháza, Hungary; 2 Department of Plant Anatomy, Institute of Biology, ELTE Eötvös Loránd University, 1117 Budapest, Hungary; katalin.solymosi@ttk.elte.hu; 3 Institute of Environmental and Natural Sciences, University of Nyíregyháza, 4400 Nyíregyháza, Hungary; richard.kovasznai.olah@gmail.com

**Keywords:** apple scions, carotenoids, chloroplasts, chlorophylls, cytokinins, plastoglobuli, thylakoid organization, ultrastructure

## Abstract

Thidiazuron, 6-benzylaminopurine riboside, and meta-topolin are cytokinins often used in apple tissue cultures. Three different CK-containing Murashige and Skoog media were used during the experiments: medium without CK or media containing 4.5 μM thidiazuron, 4.5 μM 6-benzylaminopurine riboside, or 4.5 μM meta-topolin, respectively. Comparative ultrastructural studies across cytokinin types and apple cultivars were lacking. We studied the changes in photosynthetic pigment content of the leaves with absorption spectroscopy and chloroplast structure with light and transmission electron microscopy. At the light microscopy level, large changes were detected in the length and length-to-width ratios of the chloroplasts in the spongy and palisade mesophyll cell sections in 6-benzylaminopurine riboside- and meta-topolin-treated leaves of the McIntosh scion. In the chloroplasts of the McIntosh plants treated with 6-benzylaminopurine riboside and meta-topolin, and Húsvéti rozmaring leaves treated with meta-topolin, the diameter of grana increased. In both cultivars, thidiazuron caused the height of grana to increase. Thidiazuron and 6-benzylaminopurine riboside influenced leaf anatomy both in the Húsvéti rozmaring and McIntosh cultivars. 6-benzylaminopurine riboside and thidiazuron treatments reduced the content of photosynthetic pigments in the in vitro leaves of both cultivars. In contrast, meta-topolin treatment had no significant effect on the chlorophyll content as compared to the control. Differences were observed not only among the effects of cytokinins, but even between the two apple scions examined. In in vitro apple shoot cultures, TOP maintained chloroplast integrity and pigment content, whereas TDZ exerted stress-like effects.

## 1. Introduction

Apple (*Malus x domestica* Borkh.) is a member of the *Rosaceae* family and is one of the most harvested fruit crops in the world. More than 7500 apple cultivars are cultivated in the world, differing in size, shape, color, texture, taste, and juiciness of the fruit [1]. The apple is an important part of the human diet because of its vitamin composition and mineral, antioxidant, dietary fiber, organic acids, and sugar contents [2]. Consuming apples has beneficial health effects on humans; it reduces the risk of cancer, cardiovascular diseases (heart attack, atherosclerosis), and diabetes mellitus, and it has anti-asthmatic and anti-allergic properties as well [3,4]. Tissue culture techniques have been widely used to produce clones of apple cultivars for plant production and germplasm conservation purposes.

Shoot multiplication is an important part of micropropagation in terms of productivity. It is also useful for raising virus-free plant material, for cryopreserving genetic resources, and for apple breeding through transgenics [5,6]. It is well known that many factors influence the efficiency of in vitro shoot multiplication, such as the quality of explants, composition of media, light and dark cycle, etc. However, the main factors are plant growth regulators, like cytokinins (CK) [7,8].

CKs are plant hormones that were discovered to be capable of inducing cell division. They play an important role in cell proliferation, supporting chloroplast activity and inhibiting their degradation, inducing the growth of roots and shoots, as well as inducing shoot branching [9]. In tissue cultures, plant growth regulators, such as synthetic CK- and auxin-like compounds, are used to regulate shoot and root development from various explants. In our experiments, thidiazuron (N-phenyl-N-1,2,3 thiadiazol-5-ylurea) (TDZ), 6-benzylaminopurine riboside (BAR), and meta-topolin [6-(3-hydroxybenzylamino) purine] (TOP) were used as CKs and indole butyric acid (IBA) as an auxin [6,9]. TDZ is considered to be one of the most active CK-like substances, which enhances shoot multiplication, and has been a widely used CK in apple cultures, but it has harmful side effects, such as hyperhydricity, inhibition of shoot elongation, and subsequent rooting of shoots [9].

Aromatic CKs, such as BAR and TOP, are chemical derivatives of 6-benzylaminopurine (BA); they can be used to minimize the side effects of TDZ or BA. BAR is a biologically active N^9^-riboside derivative of BA. It may cause better in vitro shoot growth in apples than BA and TDZ, but this effect is genotype-dependent. TOP is one of the most effective hydroxy-derivatives of BA and has been used in tissue cultures of certain plant species because it is free of the side effects associated with TDZ and BA [6]. The growth conditions of the in vitro tissue cultures are well controlled, and the main abiotic stressors, such as high temperature, salinity, and heavy-metal contamination, are eliminated. The most typical stress symptom is hyperhydricity, which causes apical bud and leaf top necrosis and multi-stemming [10]. Hormonal balance in tissue-cultured plants or plant parts is influenced by the type and amount of the cytokinins added to the culture medium [5,6,7]. Hormone imbalances can cause hyperhydricity in plant tissue cultures in vitro, because the level of internal CKs can be much higher than in normal tissues [11].

The response of plants to abiotic and biotic stresses can be detected at the biochemical level (e.g., as changes in enzyme activity, antioxidant capacity) and at the level of cell organelles. Under stress conditions, reactive oxygen species (ROS) are generated in mitochondria, chloroplasts, and peroxisomes of plants. Chloroplasts are one of the most studied cell components; changes in their number and ultrastructure as a result of heat, drought, salinity, and heavy-metal stress have often been reported [12,13,14]. Lipid droplets called plastoglobuli (PG) can be seen in plastids. Plastoglobuli contain carotenoids, plastoquinones, chlorophylls, or their degradation products; an increase in their number or size may indicate stress in plants [15,16]. Abiotic stress caused by the addition of plant growth regulators was also studied, for example, in barley treated with salicylic acid or in apple tissue cultures treated with BA [17,18]. In barley, at higher salicylic acid concentration, chloroplast grana stacks were lower and broader, and almost no stroma thylakoids remained in the leaves [17]. In apple tissue cultures treated with BA, the thylakoid system was dilated and strongly disturbed in the leaves [5].

Since there is a lack of systematic and comparative ultrastructural studies across CK types and cultivars, we set up the following hypotheses: (i) Thidiazuron induces stress-like alterations in chloroplast ultrastructure, but (ii) meta-topolin is able to preserve chloroplast organization and photosynthetic pigment content under in vitro conditions. Furthermore, we also hypothesized that (iii) these cytokinin-mediated effects differ between apple cultivars. Therefore, in this work, we examined the effect of BAR, TDZ, and TOP on the ultrastructure, shape, number, and size of chloroplasts, as well as chlorophyll a, chlorophyll b, total chlorophyll, and carotenoid content in the leaves of in vitro shoots of two apple cultivars (cvs. Húsvéti rozmaring and McIntosh). We also investigated whether the effects of CKs on the ultrastructure of leaf chloroplasts and photosynthetic pigment contents were cultivar-specific.

## 2. Results

### 2.1. Light Microscopy

**Figure 1:**
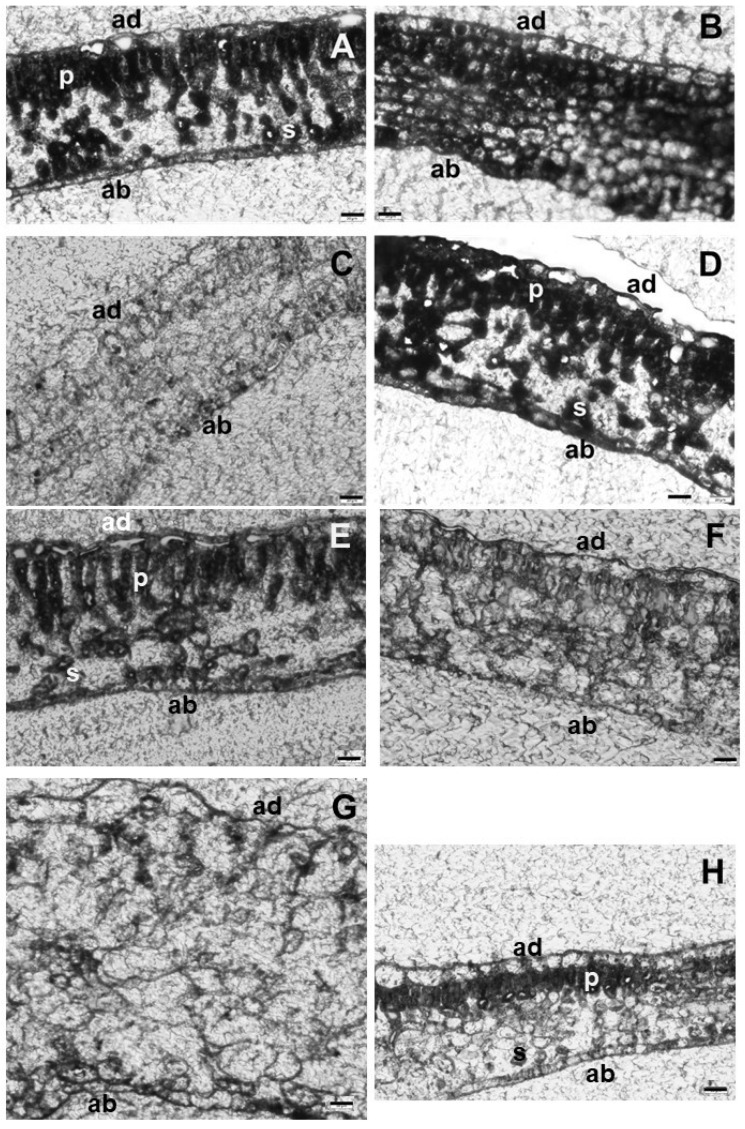
Light microscopic images of the leaf cross sections of Húsvéti rozmaring (**A**–**D**) and McIntosh (**E**–**H**) apple scion cultivars grown on medium containing no cytokinins (**A**,**E**; control) or the following cytokinins: BAR (**B**,**F**), TDZ (**C**,**G**), and TOP (**D**,**H**). p—palisade parenchyma cells, s—spongy parenchyma cells, ad—adaxial side of the leaf, ab—abaxial side of the leaf. Scale bar: 20 µm.

Light microscopic sections of the leaves of in vitro apple showed a general heterogenous mesophyll structure, with palisade parenchyma located on the adaxial side and spongy parenchyma on the abaxial side of the leaves of both apple cultivars (Figure 1A,E). In the leaves of both BAR- and TDZ-treated shoots of Húsvéti rozmaring (HR) (Figure 1B,C) and McIntosh (McI) (Figure 1F,G), adaxial and abaxial epidermises remained similar to the control leaves, but mesophyll cells became roundish, and the intercellular space decreased or disappeared. Treatment with 4.5 µM TOP had no effect on the anatomical structure of the leaves of HR (Figure 1D) or McI (Figure 1H) shoots.

In the leaves of HR and McI shoots treated with BAR and TDZ, palisade parenchyma cells were not discernible, so chloroplasts were only counted and measured in the spongy-like, rounded parenchyma cells. HR control plants had an average of 5–8 and 6–9 chloroplasts per cell section in spongy and palisade parenchyma cells, respectively. The plastid number per cell section was similar in control plants of both cultivars (Table 1). The length, the width, and the length-to-width ratio of chloroplasts were similar in these two types of mesophyll cells, with the largest difference being slightly, but not significantly, smaller plastid values observed in the palisade parenchyma cells than in spongy mesophyll cells (Table 1).

CKs had no effect on the number of chloroplasts in spongy parenchyma cells in HR; however, TDZ significantly increased, and TOP treatment significantly decreased, the plastid number in spongy parenchyma cells of McI leaves (Table 1). Plastid length significantly increased in the spongy parenchyma cells in TOP-treated leaves of HR and McI shoots (Table 1). TOP treatment also increased the plastid length in the palisade parenchyma cells in McI.

In HR plants, plastid width was not affected in any of the studied cell types, while in the spongy parenchyma cells of the leaves of McI shoots, TOP significantly increased chloroplast width (Table 1). BAR significantly increased the width of the chloroplasts in spongy parenchyma cells in McI shoots.

In both cultivars, the plastid length-to-width ratio significantly increased in spongy parenchyma cells in response to TOP treatment only (Table 1). However, in McI leaves this ratio decreased in TDZ treatment. The plastid length-to-width ratio also increased in palisade parenchyma cells of McI. No significant changes were observed in other parameters among the different treatments of the HR cultivars. Overall, plastid number and size were more affected in McI than in HR plants by CK treatments.

### 2.2. Transmission Electron Microscopy

**Figure 2:**
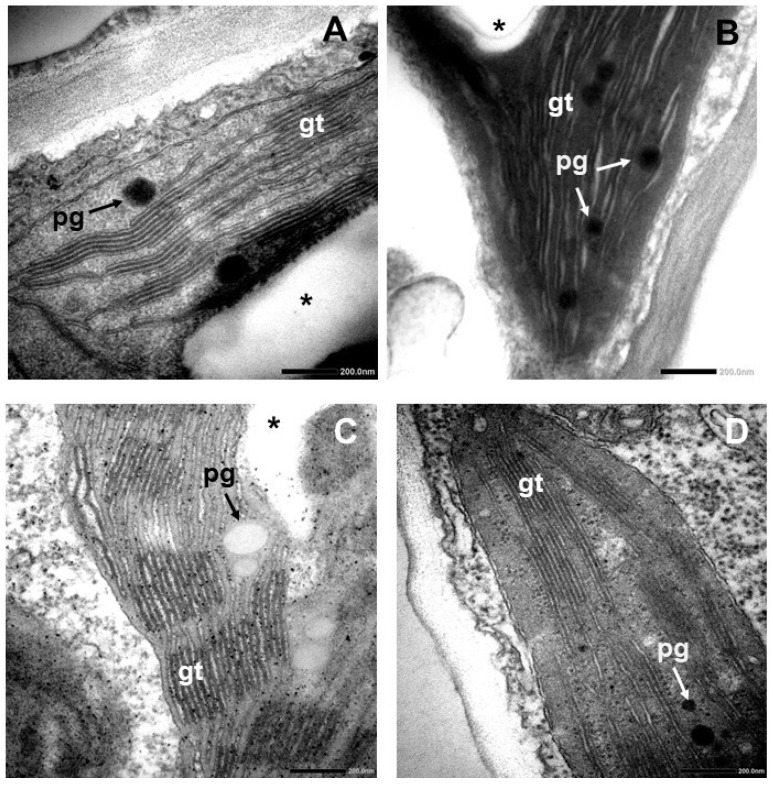
Transmission electron micrographs of typical chloroplasts in Húsvéti rozmaring apple scion cultivar grown on medium containing no cytokinins ((**A**), control) or the following cytokinins: BAR (**B**), TDZ (**C**), and TOP (**D**). Asterisk—starch grain, pg—plastoglobuli, gt—granal thylakoid. Scale bar: 200 nm.

Transmission electron microscopic (TEM) images of control and CK-treated HR apple scion cultivars showed some marked differences (Figure 2). HR control plants had regular chloroplasts with typical grana as well as well-visible thylakoids and peripheral reticulum under the inner envelope of chloroplasts. Large starch grains could be observed centrally in the chloroplast, along with electron-dense plastoglobuli (PG) in the control (Figure 2A).

**Figure 3:**
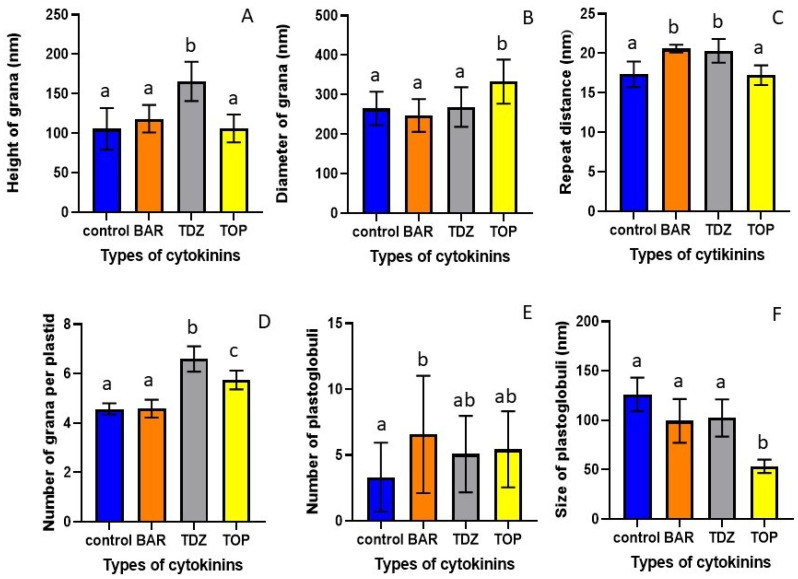
The effect of cytokinin (BAR, TDZ, or TOP) treatment on the height (**A**) and diameter (**B**) of grana, on granum repeat distance (RD) of thylakoids (**C**), on the number of grana per plastid section (**D**), on the number (**E**) and the size (**F**) of plastoglobuli per plastid section in the leaves of the HR cultivar. Different letters indicate statistically significant differences between the samples according to Kruskal–Wallis non-parametric ANOVA followed by Dunn’s multiple comparisons test (*p* < 0.05), (n = 20–25). Mean ± standard error of the mean (SEM) is presented.

The lumen of the stroma thylakoids, as well as the upper- and lowermost thylakoids of grana, were swollen; the peripheral reticulum was rarely observed, and the stroma had an unusual electron density in the chloroplasts of the leaves of HR shoots cultured on BAR-containing medium (Figure 2B). In addition, small starch grains were detected along with numerous electron-dense plastoglobuli in the plastids after BAR treatment of HR (Figure 2B and Figure 3E,F).

High, mildly swollen grana were observed in the chloroplasts of the leaves of HR shoots cultured on TDZ (Figure 2C). Larger starch grains and less electron-dense plastoglobuli were detected in the plastids after TDZ treatment of HR (Figure 2C).

Grana were similar to those in control leaves, but many transport vesicles were observed near the inner plastid envelope in the leaves of the HR shoot cultured in the presence of TOP (Figure 2D). No starch grains were present when compared to the control, but smaller plastoglobuli were noticed in plastids after TOP treatment of HR (Figure 2D and Figure 3E,F).

Quantitative analysis of granum parameters showed that TDZ treatment significantly increased the height of grana, the granum repeat distance (RD), and the number of grana per plastid section (Figure 3). BAR had a similar effect on the granum RD, while it had no effect on the other parameters (Figure 3). TOP increased the granum diameter and the number of grana per plastid section and had no effect on the height and RD of grana (Figure 3).

All CKs slightly increased the number of PG, but only BAR increased it significantly (Figure 3E). TDZ and BAR slightly, but not significantly, decreased the size of PG, while the PG size after TOP treatment was significantly smaller, less than half of the control in TOP-treated-HR leaf chloroplasts (Figure 3F).

**Figure 4:**
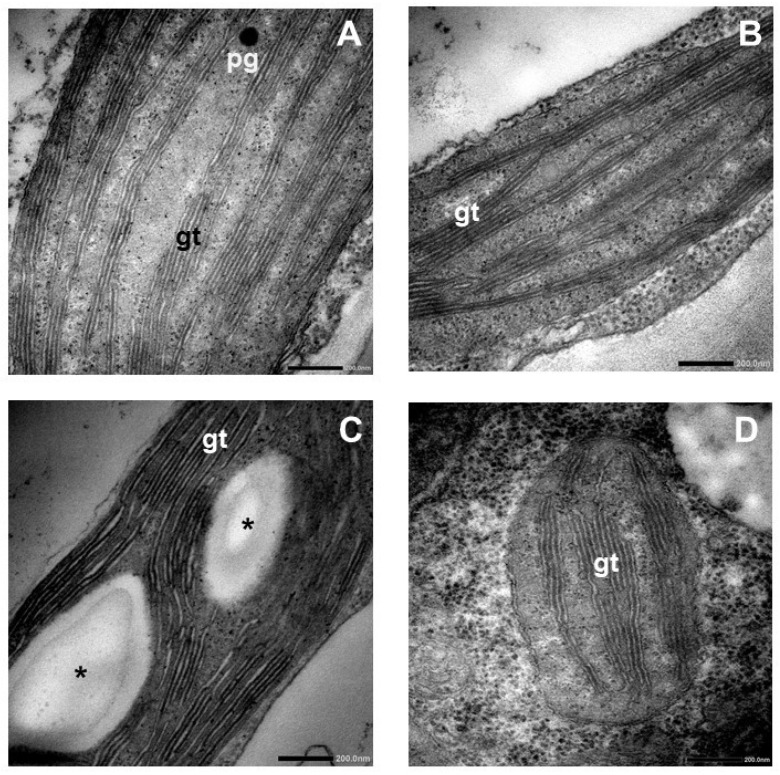
Transmission electron micrographs of typical chloroplasts in the leaves of McIntosh apple scion cultivar grown on medium containing no cytokinins ((**A**), control) or the following cytokinins: BAR (**B**), TDZ (**C**), or TOP (**D**). Asterisk—starch grain, pg—plastoglobuli, gt—granal thylakoid. Scale bar: 200 nm.

After analyzing the HR cultivar, for comparison, we carried out experiments with plants of the McI cultivar as well. Regular chloroplasts with a visible internal membrane system (stroma thylakoids and grana) were present in the control leaves of McI shoots. Small starch grains were observed centrally in the chloroplasts, along with small electron-dense plastoglobuli in the control (Figure 4A).

**Figure 5:**
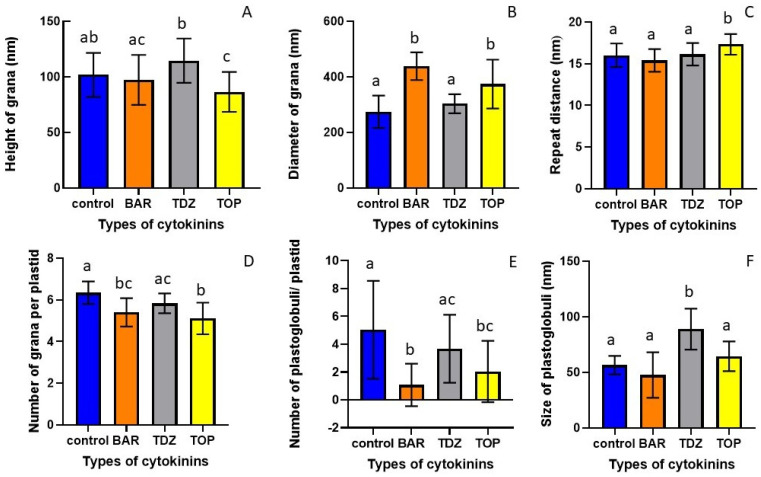
The effect of cytokinin (BAR, TDZ, or TOP) treatment on the height (**A**) and diameter (**B**) of grana, on granum repeat distance (RD) of thylakoids (**C**), on the number of grana per plastid section (**D**), on the number (**E**) and the size (**F**) of plastoglobuli per plastid section in the leaves of the McI scion cultivar. Different letters indicate statistically significant differences between the samples according to Kruskal–Wallis non-parametric ANOVA followed by Dunn’s multiple comparisons test (*p* < 0.05), (n = 20–25). Mean ± standard error of the mean (SEM) is presented.

The number of grana per plastid section was significantly lower, and granum diameter significantly increased in the leaves of McI shoots cultured on BAR compared with the control (Figure 4B and Figure 5). The peripheral reticulum was often visible in the plastids of the leaves of BAR-treated shoots, while no starch grains could be seen, and significantly fewer plastoglobuli per plastid section were detected after BAR treatment of McI (Figure 4B and Figure 5E).

Mildly swollen stroma thylakoids and grana, and as a consequence, significantly larger granum height values, as well as many transport vesicles, were observed in the plastids of the leaves of McI shoots cultured on TDZ (Figure 4C and Figure 5). Small starch grains and relatively large electron-dense plastoglobuli were detected in the plastids after TDZ treatment of McI (Figure 4C and Figure 5F).

The chloroplast ultrastructure of TOP-treated McI leaves was similar to that of control samples, and no starch grains were observed in them (Figure 4D). However, several quantitative differences could be observed. Significantly lower numbers of plastoglobuli per plastid section were observed in shoots cultured on medium containing TOP when compared to the control; however, their size had not changed (Figure 4D and Figure 5). The number of grana per plastid section was significantly lower, as well as the height of grana, while the granum diameter (not significantly lower than BAR-treated) and RD values were significantly higher in these samples than in the control (Figure 5A–C).

All CKs decreased the quantity of plastoglobuli per plastid section when compared to the control McI plants, but values in TDZ were not significantly lower (Figure 5E). TDZ increased the size of PG, while BAR and TOP had no effect on this parameter (Figure 5F).

### 2.3. Determination of the Chlorophyll and Carotenoid Content

Following acetonic extraction, we determined the photosynthetic pigment contents—chlorophyll (Chl) and carotenoid contents—of the various studied samples (Table 2). BAR and TDZ significantly decreased chlorophyll a (Chl a), chlorophyll b (Chl b), and total chlorophyll content in the leaves of both cultivars (Table 2). Carotenoid content also significantly decreased in all CK-treated HR plants, but not in McI plants. In TOP-treated HR and McI plants, Chl a, Chl b, and total chlorophyll content in the leaves were similar to those in the control. BAR, TDZ, and TOP significantly decreased the Chl a-to-Chl b ratios compared to the control in HR plants, while minor differences were observed between Chl a-to-Chl b ratios in control and TOP-treated leaves of McI shoots. BAR did not significantly increase Chl a-to-Chl b ratios, but TDZ significantly decreased them, compared to the control, in leaves of McI shoots.

## 3. Discussion

In this study, we investigated the effect of three types of cytokinins (BAR, TDZ, and TOP) on the structure of the chloroplasts in leaves of two tissue-cultured apple scion cultivars, i.e., cvs. Húsvéti rozmaring (HR) and McIntosh (McI). During our experiments, we were able to reveal the cultivar dependence of CK effects at both light and electron microscopy levels.

The anatomical structure of the leaf of both cultivars treated with TOP was similar to the control; both palisade and spongy parenchyma cells were visible (Figure 1A,D,E,H). In contrast, in the BAR- and TDZ-treated apple cultivars, only rounded parenchyma cells were visible, and palisade parenchyma cells were missing (Figure 1B,C,F,G). Similarly to these data in HR and McI cultivars, in the Royal Gala apple scion, with increased concentrations of BA treatment, the parenchyma cells became roundish [18]. We found similar changes in the anatomical structure of the leaf of BAR- and TDZ-treated HR and McI cultivars. Overall, CK types influenced the anatomical structure of the leaves in a similar manner in both cultivars. Apple leaves are hypostomatic, which means that stomata are located predominantly or exclusively on the abaxial side next to the spongy parenchyma [19]. In TDZ- and BAR-treated plants of both cultivars, the large intercellular spaces in the spongy parenchyma significantly decreased in parallel with the appearance of a homogenous mesophyll structure consisting of rounded cells at both adaxial and abaxial sides of the leaf. As apple is a typical C3 plant [20], this reorganization of the leaf anatomy might reduce gas exchange within the leaf, potentially leading to photorespiration, photoinhibition, and thus, a decline in the photosynthetic activity. However, further research would be needed to elucidate this potential question. The rounding of mesophyll cells was caused by the BAR and TDZ treatments of apple cultivars, due to the plant growth regulators themselves, while TOP did not cause these alterations of mesophyll cells. In ‘Royal Gala’, after BA treatment, mesophyll cells also became roundish [18].

At the light microscopy level, more pronounced changes were detected in the length and width of the chloroplasts in the spongy mesophyll cell sections in the leaves of McI treated with TOP than in HR (Table 1). Hyperhydricity often occurred when apple tissue culture medium was supplemented with BA, as evidenced by a decreased number of chloroplasts in palisade and spongy mesophyll cells in apple leaves [10,21]. In the leaves of HR and McI shoots treated with BAR and TDZ, palisade parenchyma cells were not observable, so chloroplasts were only counted and measured in the spongy parenchyma cells. In addition to BA, TDZ supplementation could also lead to hyperhydricity. However, TDZ had no effect on the number of chloroplasts and decreased their length in *Annona glabra* [10,22]. In contrast, BA increased the length of chloroplasts in *Triticum aestivum* [23]. In our experiments, TDZ had a similar effect on McI plants as it did on *A. glabra*, i.e., the length of the chloroplasts decreased in spongy parenchyma cells. In this work, the chloroplast number was unchanged in HR leaves throughout all applied CK treatments, while TDZ induced a significant increase in these numbers in spongy parenchyma cells of McI leaves, but TOP induced a decrease in chloroplasts per spongy parenchyma cells (Table 1). However, one should be careful with the quantitative comparison of plastid numbers per cell section because the shape and size of the mesophyll cells vary among the different samples (palisade and spongy parenchyma cells, as well as the rounded, homogenous mesophyll cells present in TDZ- and BAR-treated plants).

At the ultrastructural level, in the chloroplasts of the HR leaves treated with BAR, the number of plastoglobuli increased compared to the control, and the grana became swollen, similarly to those in the TDZ treatment, as visible on the microscopical images and also reflected in the increase in granum RD values (Figure 2B and Figure 3). Muniz de Oliveira et al. [22] also found large plastoglobuli in TDZ-treated *A. glabra*. We observed similar changes, i.e., an increase in plastoglobule size in the leaf chloroplasts of TDZ-treated McI shoots (Figure 5). In the chloroplasts of HR shoots, TOP treatment significantly decreased the plastoglobule size (Figure 3F). The enlargement of plastoglobule size is often observed under various stress conditions, such as nitrate starvation, drought, or high saline concentration [24,25,26,27,28]. Alterations in the plastoglobuli size and number of leaves in CK-treated HR and McI shoots may be due to the imbalance of endogenous hormones caused by the addition of exogenous cytokinins to the tissue culture medium.

After TDZ treatment of both HR and McI shoots, mildly swollen grana were visible in leaf chloroplasts on the microscopic images and reflected in the increase in granum RD values (Figure 2, Figure 3, Figure 4 and Figure 5). Swollen grana can be observed in tissue cultures of other species (e.g., *Dianthus caryophyllus*, *A. glabra*) after treatment with BA and TDZ, resulting in hyperhydricity [10,21,22]. In Royal Gala apple scion shoots grown in vitro on medium containing BA, the thylakoid system was dilated and disturbed in the chloroplast [5,18]. When the medium contained TOP, the ultrastructure of the chloroplast was regular and lacked starch grains [24]. The ultrastructure of the chloroplasts was also regular in the leaves of the HR and McI apple cultivars treated with TOP in the present experiment. Similarly to the data obtained from the Royal Gala cultivar, no starch grain was observed. During plant growth and development, CKs play an important role in the differentiation of proplastids into chloroplasts and the transition of etioplasts into chloroplasts in plants grown in the dark that are then transferred to light. However, exogenous CKs can also alter the ultrastructure of chloroplasts in plants grown in light [29,30,31,32,33]. These changes can be observed as an increased number of thylakoids per grana or as swollen grana [29,30]. The swollen grana, as observed in the leaves of TDZ-treated HR and McI shoots, were similar to what was previously described in the BA-treated Royal Gala, and may be a side effect of culturing in vitro apple shoots on medium containing TDZ or BA. The regular ultrastructure of the chloroplasts, observed in the TOP-treated HR and in the BAR- and TOP-treated McI plants, is similar to that described earlier in the TOP-treated Royal Gala. This is likely due to the fact that BAR and TOP have fewer side effects in apple tissue culture than TDZ does [6,30,31].

Differences related to different types of CKs were observed in both apple cultivars. The differences in characteristics of grana and plastoglobuli, as well as the presence or absence of starch grains between the two cultivars, are due to the differences in the responses of the cultivars to various CKs. CKs may increase the starch content of chloroplasts in addition to affecting on number of thylakoids per grana [30]. However, there were no starch grains in the leaf chloroplasts of BAR-treated McI and TOP-treated HR and McI shoots.

Interestingly, the photosynthetic pigment content of McI leaves was significantly lower than that of the HR cultivar (Table 2). All CK treatments decreased the Chl a-to-Chl b ratio in the leaves of HR shoots, while only the TDZ treatment decreased it significantly in the leaves of McI shoots. The TOP-treated McI and HR leaves had the highest Chl a and b content, as well as the total chlorophyll and carotenoid content among the CK-treated plants. The lowest level of Chl a was found in the leaves of the TDZ-treated McI and HR plants (Table 2). These results are in accordance with the similar findings in the Royal Gala cultivar [34]. Amoo et al. [35] observed a similar effect of BAR and an opposite effect of TOP on Chl a and b, as well as the total Chl and carotenoids in *Aloe arborescens* Mill. Amoo et al. [36] observed similar effects of TDZ and the opposite effect of TOP on Chl a and b, as well as the total Chl and carotenoid content in *Merwilla plumbea* (Lindl.) Speta. TOP increased Chl content in *Sesamum indicum* L. and *Oxystelma esculentum* (L. f.) Sm. [37,38,39].

The practical benefit of our experimental results in apple tissue culture is that it strengthens the importance of TOP as a superior cytokinin for maintaining functional chloroplasts in apple micropropagation.

## 4. Materials and Methods

### 4.1. Plant Materials, Growing Conditions, and Collecting of Plant Samples

Plant material was collected from shoots of four-week-old in vitro shoot cultures of two *Malus x domestica* Borkh. scion cultivars McIntosh and Húsvéti rozmaring. Shoot cultures were maintained and subcultured monthly on Murashige and Skoog (MS) basal medium, pH 5.8 [40], supplemented with 2.22 μM 6-benzylaminopurine (BA), 0.49 μM indole butyric acid (IBA), 0.58 μM gibberellic acid (GA_3_), 3% (*w*/*v*) sucrose, and 0.7% (*w*/*v*) agar-agar. Each culture vessel contained 70 mL of medium and five shoot explants. Shoot cultures were grown at 23 ± 2 °C under 16/8 photoperiod, at light intensity of 80–106 μmol s^−1^m^−2^. Prior to the experiments, shoots were grown for four weeks in vitro on CK-free medium to avoid the after-effects of CK in the experiments.

Four-week-old in vitro shoots grown on CK-free medium were separated from in vitro mother shoots, cut into 20 mm long segments, and placed on experimental media. Except for the CK content, the experimental media were the same as the medium used for maintenance described above. Four different CK-containing media were used during the experiments: medium without CK (control) and media containing 4.5 μM TDZ (N-phenyl-N-1,2,3 thiadiazol-5-ylurea), 4.5 μM BAR, or 4.5 μM TOP [6-(3-hydroxybenzylamino) purine)], respectively. Culture conditions were the same as those applied for the maintenance of shoot cultures. From the four different culture vessels for each of the experimental CK treatment cultures, the third fully developed leaf from the top of each of the five shoots was collected at the end of the fourth week (i.e., end of subculture) for microscopic studies.

### 4.2. Light Microscopy

To analyze leaf anatomy, samples were taken from the leaf blade. Segments of 2 × 1 mm, located at the center part of the leaf blades, but at least in a 1 mm distance from the major vein, as well as 3–3 mm from the apex and base of the leaf, respectively, were collected in freshly made solution of 0.075 mM Na phosphate (pH 7.2), 4.0% (*V*/*V*) formaldehyde, and 2.5% (*V*/*V*) glutaraldehyde solution for fixation for at least 3 h. Rinsing after the primary fixative solution with buffer was followed by post-fixation in osmium-tetroxide, then rinsing of the post-fixative with buffer and dehydration of the samples in acetone. Finally, samples were embedded in Durcupan ACM (Fluka, Buchs, Switzerland), and thin (3–4 µm) sectioning was carried out. Sections were stained with toluidine blue and studied using a light microscope. Digital light microscopic images were captured using an Olympus BX43 fluorescence microscope (Tokyo, Japan). The size (length and width) of the chloroplasts was measured, and the number of chloroplasts per mesophyll cell section was determined on these sections. For image analysis, Image J v 2.16.0 software was used.

### 4.3. Transmission Electron Microscopy

After thin sectioning, 70 nm ultrathin sections were cut from the samples fixed for light microscopy and contrasted with lead citrate before being analyzed with a JEM-1400 FLASH (JEOL, Tokyo, Japan) transmission electron microscope. Diameter and number of plastoglobuli were determined in mesophyll cells. Height and diameter of grana, number of grana per plastid section, and number and size of plastoglobuli were determined on 20–25 randomly chosen chloroplasts. For image analysis, Image J v 2.16.0 software (NIH, Bethesda, MD, USA) was used. Fast Fourier transformation (FFT) was performed on the selected regions of interest (i.e., grana) of micrographs using Image J (NIH, Bethesda, MD, USA) software to determine the granum repeat distance (RD) values on them according to Ünnep et al. [41].

### 4.4. Determination of Chlorophylls and Carotenoids

The chlorophyll and carotenoid contents were determined in the leaves of four-week-old tissue-cultured shoots. For this assay, 100 mg of fresh tissue culture was ground with 5 mL of 80% acetone, and after which, the sample was centrifuged at 2500 RPM for five minutes. The absorbance of the supernatant was measured at 660, 645, and 470 nm. The chlorophyll a (Chl a), chlorophyll b (Chl b), and carotenoid content were determined in milligrams per gram of fresh weight (mg/g FW) using the following equations according to Arafa et al. [42]. A660, A645, and A470 are the respective absorbance values, V is the volume of the extract, and FW is the weight of the fresh tissue culture.

$$Chl\;a=\frac{\left[12.21\left(A660\right)-2.81\left(A645\right)\right]V}{\left(1000FW\right)}$$

$$Chl\;b=\frac{\left[20.13\left(A645\right)-5.03\left(A660\right)\right]V}{\left(1000FW\right)}$$

$$Carotenoid=\frac{\left[1000\left(A470\right)-23.27\left(Chla\right)-104(Chlb)\right]V}{\left(1000FW\right)}$$

### 4.5. Statistical Analyses

Statistical analyses (normality test, ANOVA, and post hoc tests) were performed using GraphPad Prism 8 (GraphPad Software, La Jolla, CA, USA). When the data followed normal distribution, and significant differences were detected by one-way ANOVA, the Tukey–Kramer multiple comparisons test was used as a post hoc test. For data that did not follow a normal distribution, the Kruskal–Wallis non-parametric ANOVA test was performed. This was followed by Dunn’s multiple comparisons test as a post hoc test. Significant differences were labeled with different letters. For all data, *p* < 0.05 was considered significant. For the light microscopic determination of the number of chloroplasts per mesophyll cell, the length, the width, and the length-to-width ratio of chloroplasts, 25 cells and 25 chloroplasts were sampled randomly across multiple leaves in each treatment. To determine the height and diameter of grana, the number of grana per plastid section, and the number and size of plastoglobuli, 20–25 chloroplasts were sampled randomly across the leaf mesophyll from different sections across a single leaf in transmission electron microscopy. Approximately 6–8 grana were randomly measured from each chloroplast, and from these, the RD values were averaged from 120 to 170 grana. For the determination of the pigment content data, each data point represented a pooled sample consisting of 100 mg of leaf tissue collected from five shoots present in the same culture vessel. Plants from four parallel-grown culture vessels were measured within each independent experiment, and these experiments were repeated independently three times.

## 5. Conclusions

In the present experiments, we observed differences in the effects of various CKs applied in tissue culture medium on the structure of leaf chloroplasts, as well as chlorophyll and carotenoid content of the leaves of two different apple cultivars. Furthermore, we revealed that ultrastructural responses to CKs and the photosynthetic pigment content varied depending on the cultivar. TOP treatment decreased the height of grana in HR, but had no effect on it in McI. BAR treatment had no effect on the diameter of grana in HR, while it increased it in McI. The repeat distance increased only after BAR and TDZ treatment; TOP had no effect on it in HR, however, only TOP treatment increased it, and BAR and TDZ had no effect in McI. TDZ and TOP treatment increased the number of grana in HR, but all the CKs decreased it in McI. All the CKs increased the number of plastoglobuli in HR, but all of them decreased it in McI. TOP decreased the size of plastoglobuli in HR, while TDZ increased it in McI. In most cases, TDZ had negative effects, such as decreasing photosynthetic pigment concentration and negatively affecting the ultrastructure of chloroplasts. One possible reason for this was that TDZ caused higher oxidative stress in tissue cultures than BAR or TOP did [43,44,45]. BAR and TOP had similar effects on the photosynthetic pigment content of HR and McI leaves, which might not be surprising as they have the same benzylaminopurine backbone. However, the activity of the CK depends on the side chain. Hydroxylated derivatives (TOP) are much more active than ribosides (BAR) [46,47], which may explain the differences detected between the effects of BAR and TOP on the ultrastructure and photosynthetic pigment content of the leaves. The novelty of our experiment is that we studied and compared the effects of BAR, TDZ, and TOP treatment on the chloroplast ultrastructure, leaf anatomical structure, and photosynthetic pigment content in in vitro shoot cultures of two apple scions. In summary, it can be concluded that TDZ induced stress-like remodeling of chloroplast structure, whereas TOP preserved chloroplast ultrastructure and photosynthetic pigment integrity. The observed cultivar-specific responses highlight the importance of genotype-dependent optimization of in vitro culture protocols [5,18,21,22].

## Figures and Tables

**Table 1:** Effect of cytokinin (BAR, TDZ, or TOP) treatment on the number of chloroplasts per plastid section, on the length, the width, and the length-to-width ratio of chloroplasts in different cell layers (e.g., palisade and spongy parenchyma) of the leaves of Húsvéti rozmaring (HR) and McIntosh (McI) apple scions. Different letters in each row indicate statistically significant differences between the samples according to Kruskal–Wallis non-parametric ANOVA followed by Dunn’s multiple comparisons test (*p* < 0.05). Mean values and standard error of mean are presented (n = 25). ND—not determined.

	Húsvéti Rozmaring (HR)				McIntosh (McI)			
	Control	BAR	TDZ	TOP	Control	BAR	TDZ	TOP
Number of plastid/spongy parenchyma cells	6.40 ± 0.4 a	5.96 ± 0.2 a	6.4 ± 0.3 a	5.84 ± 0.3 a	6.40 ± 0.2 a	6.32 ± 0.2 ac	7.48 ± 0.2 b	5.28 ± 0.3 c
Number of plastids per palisade cell	6.68 ± 0.3 a	ND	ND	6.32 ± 0.3 a	6.84 ± 0.2 a	ND	ND	7.68 ± 0.4 a
Plastid length in spongy parenchyma (µm)	2.39 ± 0.1 a	1.99 ± 0.1 a	1.93 ± 0.1 a	3.40 ± 0.1 b	2.28 ± 0.2 a	3.52 ± 0.2 b	1.65 ± 0.1 a	3.68 ± 0.1 b
Plastid length in palisade parenchyma (µm)	2.11 ± 0.1 a	ND	ND	1.99 ± 0.1 a	2.27 ± 0.2 a	ND	ND	3.37 ± 0.1 c
Plastid width in spongy parenchyma (µm)	1.85 ± 0.1 a	1.70 ± 0.1 a	1.56 ± 0.1 a	1.80 ± 0.1 a	1.29 ± 0.1 a	2.11 ± 0.1 b	1.31 ± 0.1 a	1.68 ± 0.1 c
Plastid width in palisade parenchyma (µm)	1.70 ± 0.1 a	ND	ND	1.44 ± 0.1 ab	1.54 ± 0.1 a	ND	ND	1.44 ± 0.1 ac
Plastid length-to-width ratio in spongy parenchyma	1.35 ± 0.1 a	1.20 ± 0.1 a	1.25 ± 0.1 a	2.05 ± 0.2 b	1.77 ± 0.1 a	1.69 ± 0.1 a	1.28 ± 0.1 b	2.16 ± 0.1 c
Plastid length-to-width ratio in palisade parenchyma	1.27 ± 0.1 a	ND	ND	1.43 ± 0.1 a	1.49 ± 0.1 a	ND	ND	2.30 ± 0.1 b

**Table 2:** Effect of different cytokinins (BAR, TDZ, and TOP) treatment on chlorophyll a (Chl a), chlorophyll b (Chl b), total chlorophyll, chlorophyll a-to-chlorophyll b ratio (Chl a-to-Chl b ratio), and total carotenoid in the leaves of Húsvéti rozmaring (HR) and McIntosh (McI) apple scions. Different letters in each row indicate statistically significant differences between the samples according to Kruskal–Wallis non-parametric ANOVA followed by Dunn’s multiple comparisons test (*p* < 0.05). Mean values and standard error of mean are presented, n = 12.

	Húsvéti Rozmaring (HR)				McIntosh (McI)			
	Control	BAR	TDZ	TOP	Control	BAR	TDZ	TOP
Chl a (mg/g FW)	2.37 ± 0.10 a	0.89 ± 0.01 b	0.74 ± 0.02 b	2.25 ± 0.03 a	1.74 ± 0.10 a	1.21 ± 0.10 b	1.01 ± 0.02 b	1.73 ± 0.10 a
Chl b (mg/g FW)	0.93 ± 0.01 a	0.45 ± 0.02 b	0.38 ± 0.03 b	1.12 ± 0.03 a	0.76 ± 0.02 a	0.47 ± 0.02 b	0.57 ± 0.02 c	0.81 ± 0.02 a
total Chl (mg/g FW)	3.29 ± 0.02 a	1.34 ± 0.03 b	1.12 ± 0.10 b	3.36 ± 0.05 a	2.50 ± 0.10 a	1.67 ± 0.10 b	1.57 ± 0.04 b	2.54 ± 0.10 a
Chl a-to-Chl b ratio	2.56 ± 0.04 a	1.99 ± 0.10 b	2.01 ± 0.10 b	2.02 ± 0.04 b	2.29 ± 0.10 a	2.59 ± 0.10 a	1.80 ± 0.10 b	2.15 ± 0.10 ab
carotenoid (mg/g FW)	0.18 ± 0.01 a	0.06 ± 0.01 b	0.06 ± 0.01 b	0.16 ± 0.01 b	0.12 ± 0.01 a	0.08 ± 0.01 ab	0.06 ± 0.01 ab	0.14 ± 0.01 ac

## Data Availability

The original contributions presented in this study are included in the article. Further inquiries can be directed to the corresponding authors.
